# Balloon Kyphoplasty Complication: A Case of a Lodged Balloon Within the Vertebral Body

**DOI:** 10.7759/cureus.10542

**Published:** 2020-09-19

**Authors:** Grace Chalhoub, Brian Cheung, Christian Gonzalez

**Affiliations:** 1 Osteopathic Medicine, Nova Southeastern University, Davie, USA; 2 Anesthesiology, Aventura Hospital and Medical Center, Aventura, USA; 3 Anesthesiology, Kendall Regional Medical Center, Miami, USA; 4 Pain Management, Spine and Wellness Centers of America, Miami, USA

**Keywords:** kyphoplasty, compression fracture, osteoporosis, spine, balloon, cement extravasation, bone tamp

## Abstract

This report describes a case involving a balloon kyphoplasty bone tamp becoming lodged inside the vertebral body and unable to be withdrawn, the first report of its kind in the literature. A board certified interventional pain management physician was performing a balloon kyphoplasty for an L3 osteoporotic vertebral compression fracture using a bipedicular approach with two bone tamps. Cannulation and cavity formation were completed without complication; however, upon removal of the balloons it was noted that one had become lodged in the vertebral body. Several attempts were made to remove the balloon. Neurosurgery and the balloon manufacturer were consulted intraoperative, and it was decided to leave the balloon fragments in situ and complete the interventional fixation of the vertebral body with bone cement. The patient followed up in the clinic several months later without neurologic complications. Postoperative radiography confirmed the presence of a retained foreign body consistent with balloon fragments. Balloon kyphoplasty and its various procedural complications will be discussed, as well as the intraoperative decision making faced when encountering a complication.

## Introduction

Balloon kyphoplasty (BKP) is a minimally invasive, percutaneous procedure used to restore vertebral body height and repair spinal compression fractures caused by osteoporosis. BKP is indicated for acute symptomatic compression fractures with height loss of >20%. It is a type of vertebroplasty utilizing an inflatable bone tamp in the vertebral body to restore vertebral height prior to injection of bone cement. Similar to traditional vertebroplasty, BKP presents with many complications. Those complications include cement extravasation, thermal injury and post-procedural pain. However, BKP has been shown to be more effective at restoring vertebral height at lower pressures and with fewer complications than traditional vertebroplasty. BKP has some complications specific to it, such as further vertebral fracture from balloon expansion, balloon rupture, and re-collapse following balloon deflation. This report describes a BKP in which the bone tamp broke off inside the patient and was unable to be removed, the first case of which to be described in the literature.

## Case presentation

**Figure 1:**
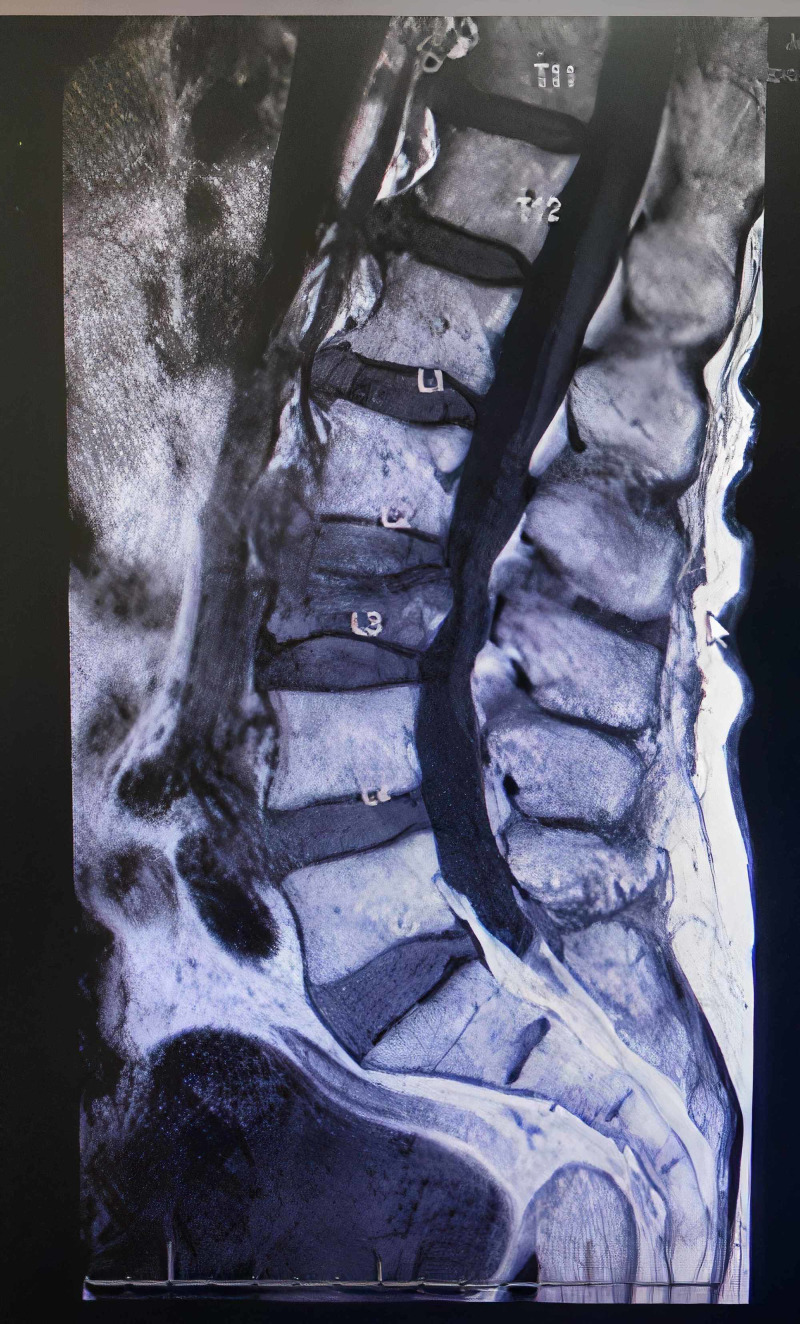
Preoperative MRI, sagittal plane, showing subacute L3 compression fracture with a 50%-60% loss of height

A 83-year-old male patient presented to pain management with low back pain ongoing for several weeks. The patient was previously seen by an orthopedic surgeon who recommended a conservative treatment with the thoracic lumbosacral orthosis (TLSO) brace with which the patient was non-compliant. Multiplanar multisequence non-contrast MR images of the lumbar spine were obtained (Figure 1). Findings were positive for a subacute compression fracture of the L3 vertebral body with a 50%-60% loss of height and mild bilateral foraminal stenosis at L4-L5.

**Figure 2:**
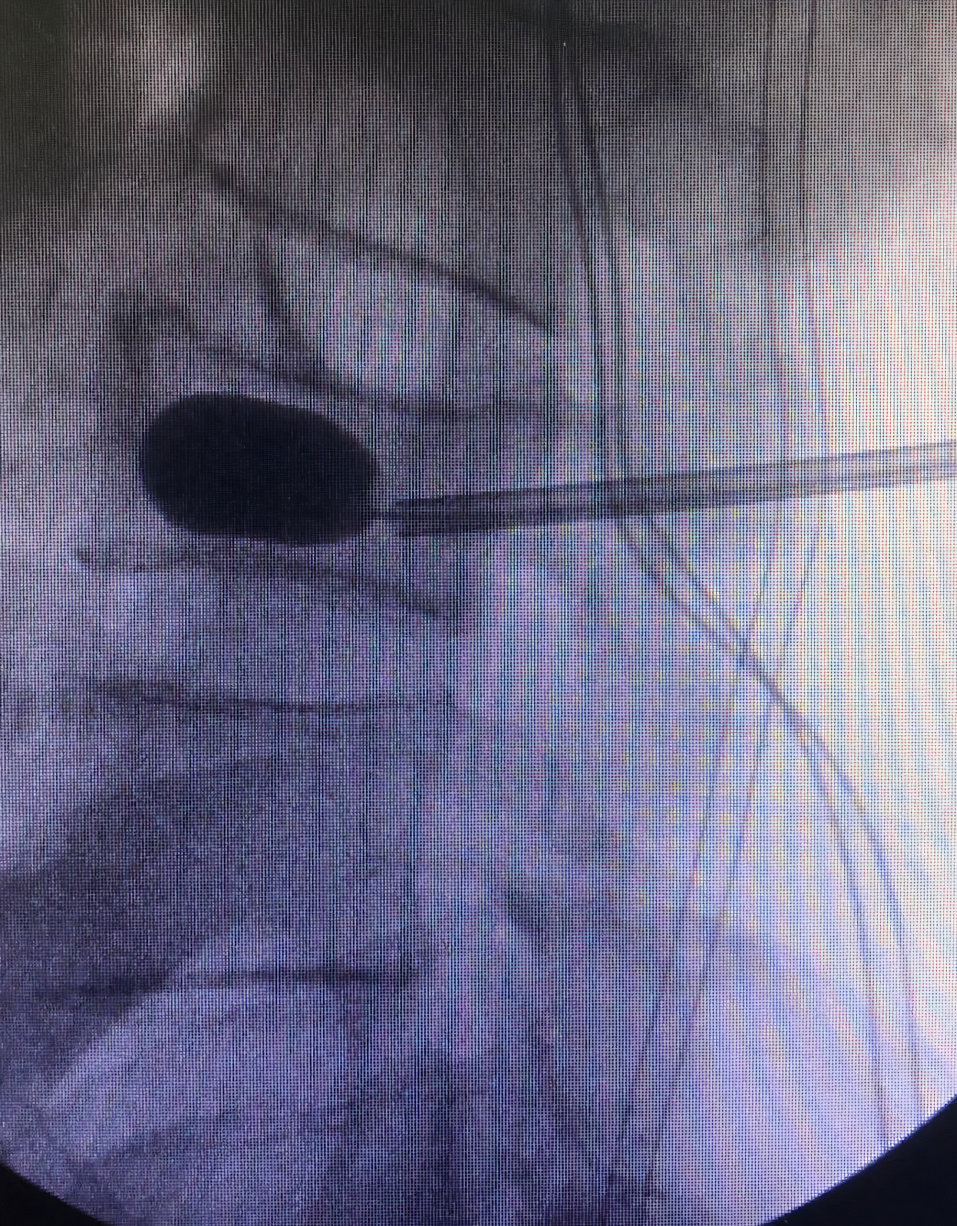
Intraoperative fluoroscopy, lateral image showing balloon inflation and restoration of height

**Figure 3:**
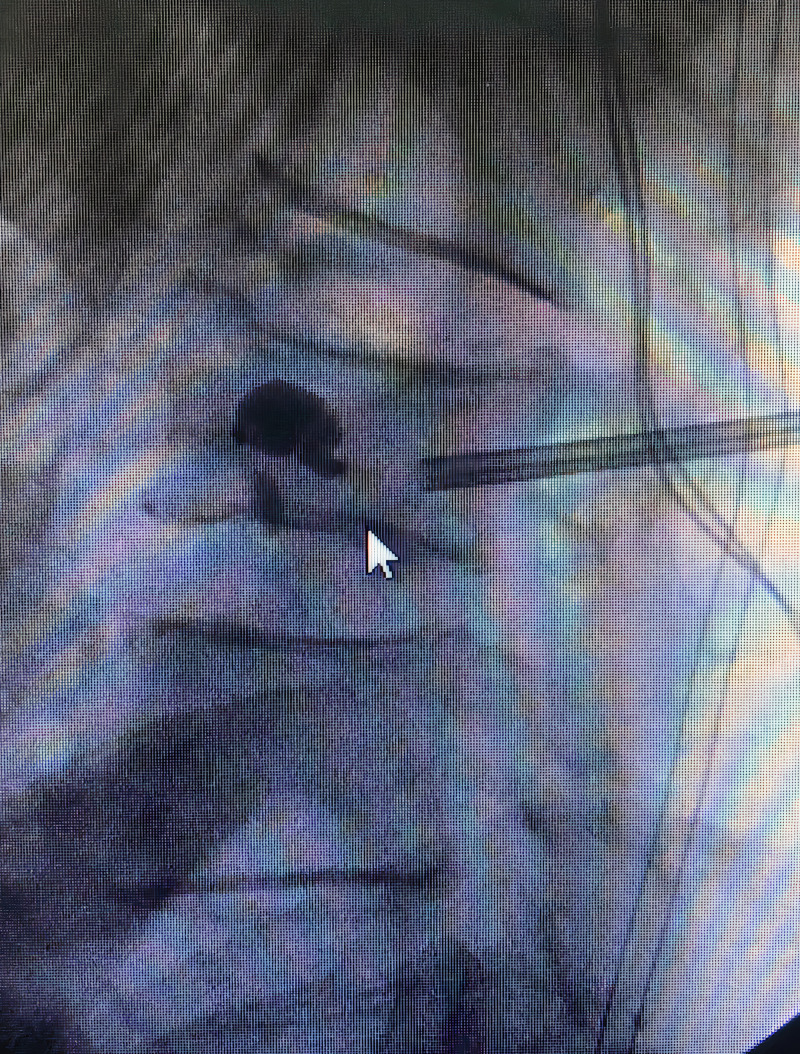
Intraoperative fluoroscopy, lateral image showing retained foreign body suspicious for broken balloon

The patient was offered BKP for correction of vertebral kyphosis and symptomatic relief. A BKP was performed with re-expansion of the vertebral body under fluoroscopic guidance and injection of bone cement (Figure 2). Following removal of the catheters, a radiopaque foreign body was visualized along the posterior vertebral body, suspicious for a retained balloon marker (Figure 3).

## Discussion

Vertebroplasty was first described in 1984 in France for the treatment of a vertebral hemangioma and involves a transpedicular approach with infiltration of polymethylmethylacrylate under pressure to stabilize the bone. Vertebroplasty has shown improved quality of life and pain scores up to one year following the procedure when compared with non-surgical management. Both traditional vertebroplasty and BKP show an improvement in kyphotic angle following the procedure with BKP maintaining results for up to three years [1-4]. When compared with traditional vertebroplasty, BKP allows for greater restoration of vertebral height and cement injection at lower pressures with fewer complications. Complications common to both include cement extravasation, thermal injury during the curing process, post-procedural pain, and new adjacent vertebral fractures due to altered loading stresses on the spinal column [5]. Complications specific to BKP include further vertebral fracture from balloon expansion, balloon rupture, and re-collapse following balloon deflation [6].

Following review of the literature, this is the first report of a retained bone tamp following BKP. There are many possible factors that may have contributed to the shearing and retention of the balloon. Examples of such factors include the pressure used in order to inflate the bone tamp [7], an acute angle of the balloon to the cannulae, a balloon incompletely deflated prior to removal, the intrinsic physical properties of the balloon, and a sharp bone fragment in close proximity to the balloon that can rupture it even at low pressures [8]. SpineJack® kyphoplasty is a newer kyphoplasty technique that does not require the use of a balloon and therefore eliminates the risk of balloon-related complications. It has also shown to be more effective at vertebral height restoration [9].

## Conclusions

BKP generally presents with fewer complications than traditional vertebroplasty. However, it is not denuded of complications such as further vertebral fracture from balloon expansion, balloon rupture, and re-collapse following balloon deflation. A retained bone tamp is now a new reported complication. 
